# Migration-Selection Balance at Multiple Loci and Selection on Dominance and Recombination

**DOI:** 10.1371/journal.pone.0088651

**Published:** 2014-02-14

**Authors:** Alexey Yanchukov, Stephen R. Proulx

**Affiliations:** Department of Ecology, Evolution and Marine Biology, University of California Santa Barbara, Santa Barbara, California, United States of America; Institut Jacques Monod, France

## Abstract

A steady influx of a single deleterious multilocus genotype will impose genetic load on the resident population and leave multiple descendants carrying various numbers of the foreign alleles. Provided that the foreign types are rare at equilibrium, and all immigrant genes are eventually eliminated by selection, the population structure can be inferred explicitly from the branching process taking place within a single immigrant lineage. Unless the migration and recombination rates were high, this novel method gives a close approximation to the simulation with all possible multilocus genotypes considered. Once the load and the foreign genotypes frequencies are known, it becomes possible to estimate selection acting on the invading modifiers of (i) dominance and (ii) recombination rate on the foreign gene block. We found that the modifiers of the (i) type are able to invade faster than the type (ii) modifier, however, this result only applies in the strong selection/low migration/low recombination scenario. Varying the number of genes in the immigrant genotype can have a non-monotonic effect on the migration load and the modifier’s invasion rate: although blocks carrying more genes can give rise to longer lineages, they also experience stronger selection pressure. The heaviest load is therefore imposed by the genotypes carrying moderate numbers of genes.

## Introduction

Gene flow is a major force shaping the evolution of closely related sexual populations, which can prevent local adaptation [1], [2] or facilitate generalism [3], [4], erase or maintain intraspecific polymorphism [5], [6], promote the evolution of female mating preferences [7], [8] and lead to speciation through reinforcement [9], [10]. Such diverse evolutionary outcomes arise because the exchange of genetic material generates variance and so is intervened by some form of selection: in a vast number of studied cases, selection affects only a discrete set of genes, small in proportion to the individual genome size [11]–[13]. When individuals of foreign origin make their first entry into the resident population, they carry an intact set of genes (i.e. in complete linkage disequilibrium), but after repeated backcrosses with the resident genotypes (i.e. introgression), there will be multiple descendants of the first migrants carrying some proportion of the initial foreign genotype. Because natural selection acts on the individual level, the actual selective pressure on each foreign gene will depend on its association with the other selected loci: in the most common scenario where the foreign genes are deleterious, the first generation hybrids with high LD will be strongly selected against, while the backcrosses will experience less selective pressure [14].

The interplay between gene flow and selection thus leaves a specific signature on the genetic structure of the resident population, observed, for example, in many hybrid zones [15] and subject to extensive applied modeling aimed primarily at the data analysis [16]. More rigorous theoretical treatment of the introgression, is, however, difficult since too many genotypes have to be considered even for a moderate number of loci: significant progress in this area has been made but involves a great deal of approximation and simplifying assumptions about the nature of recombination of the foreign set of genes [17], [18]. Here, we develop this theory further and propose a new simple model of gene flow and selection that allows for an explicit characterization of the resident population structure, provided that the population is large, migration rate is low and the foreign genes are evenly distributed in a linear genome block.

Under the premise that diversifying selection is a major factor responsible for the differences between closely related populations, one expects that genes brought into the new environment (including new genomic environment) will experience negative selection pressure [19]. This means that all *selected* introgressed material descending from the first immigrant individual will almost certainly disappear as time progresses, but until then, the mean fitness of the resident population will remain suboptimal. We show that it is possible to calculate the reduction in population mean fitness caused by the immigrant lineage throughout its path to extinction. In fact, this measure turns out to be equal to the actual migration load imposed on the population by gene flow and calculated at any time point once the migration-selection process is at equilibrium, because immigrant lineages, arriving at a slow rate, will segregate *independently* in a large resident population. Based on the same principle, we derive the equilibrium frequencies of genotype classes carrying the same numbers of the introgressing genes, and thus arrive at a good approximation of the population genetic structure. Once the population structure corresponding to a given point in parameter space is known, we can estimate the strength of selection favoring specific microevolutionary mechanisms that render the migration-selection balance unstable [20].

What theoretical consequences can follow from the equilibrium between multilocus gene flow and selection? Since mean population fitness is reduced, any novel mechanism that allows it to recover towards the maximal level should be favored by selection [21], [22]. Obviously and trivially, a reduction in the migration load would be achieved simply by decreasing gene flow, for example by reducing the number of migrants between demes [23]. However, this could also be achieved by selection on mating preferences [7], [22], [24]. Here we add to the vast body of literature on this subject by asking what level of selection pressure will act on a modifier that (i) masks the deleterious effect of the immigrant genes, therefore increasing the robustness of the resident genotype to the gene flow and (ii) suppresses recombination between the foreign genes, thus increasing the efficacy of selection on multiple loci. Canalization of deleterious alleles is widespread among eukaryotes, particularly in the form of diploid dominance, and a few theoretical studies demonstrated that either of these can evolve in response to migration [25], [26]. Genomic clustering of the adaptive loci has also been shown to evolve under the migration-selection balance [27], [28]. In this article, we demonstrate that the corresponding dominance or recombination modifiers can increase in frequency under comparable conditions, and that the amount of selection acting on them can be significant relative to the migration load [21].

## Results

### The General Model: Multilocus Introgression at the Migration-selection Equilibrium

**Figure 1:**
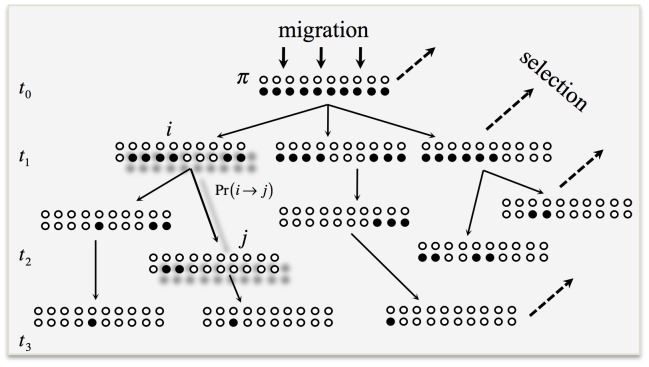
A subset of the lineage descending from an immigrant haplotype 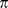
 **carrying 10 genes.** After selection, the genotypes carrying foreign genes (solid black dots) recombine with the resident genotypes (shown as the rows of open dots), so that the average number of foreign alleles per genotype is reduced every generation 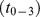. Once recombination has broken the foreign genotype down to one gene, it is certain to eventually be eliminated by selection in the single foreign allele state.

Our basic model is a generalization of Barton’s [17] model of low rate gene exchange between two demes. Consider a large population in which a small proportion *m* of residents is replaced by an equal fraction of immigrants each generation. All immigrants have the same genotype consisting of a finite number of discrete genes, that is, the genomic distance between any two genes far exceeds the size of the gene itself, and recombination is unlikely within the gene. Following migration, there is direct selection against the foreign genotypes in the resident population, which generally becomes weaker as the average number of the foreign genes per individual decreases due to recombination, but remains strong enough to counter-balance gene flow and maintain a migration-selection equilibrium. The total fraction of recombinant individuals is still assumed to be small even after the equilibrium is reached, so that we can safely ignore unions between gametes that both carry foreign genes. Selection is therefore only acting on heterozygotes: even if the immigrant individuals were homozygous, their progeny will be heterozygous after just one round of selection. The population at equilibrium is characterized by the mean population fitness 
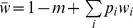
, estimated at the haploid stage, where 1−*m* is the resident genotype frequency after migration, and 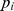 is the frequency of the foreign genotype *i* weighted by its relative fitness 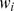. Due to migration and selection, 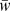 is less than the fitness of the resident genotype 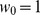, imposing the migration load 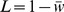 on the population. Let 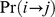 be the unspecified (for now) probability of transition between the non-identical genotypes labeled *i* and *j*, carrying the numbers *k* and *l* of foreign genes, respectively, which we will call the *lengths* of the genotypes. Note that because immigrants only mate with the residents, *j* cannot carry more foreign genes than *i* (i.e. 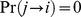, for any *j*>*i*), and let 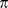 be the initial immigrant type, which has the maximum length 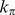 (Fig. 1). Over time, this transition probability will completely determine the distribution of genotypes within a set of individuals sharing a common ancestry and carrying the introgressed genetic material, which we will call a *lineage* [29]. Since the transition probability does not change through time, and there is no direct interaction between the foreign genotypes that belong to different lineages, the dynamics of the population can be classified as the multi-type branching process [29]–[31]. We will now demonstrate that the knowledge of the mechanisms of selection and recombination that take place within a single lineage is also sufficient to characterize the genetic structure of the population at migration-selection equilibrium. First, the mean population fitness 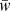 can be expressed alternatively as:
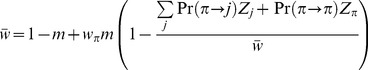
(1)


The term 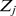, which we will call the relative *size* of the lineage *j*, represents the contribution to the mean fitness made by all future descendants of the genotype 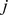. Correspondingly, 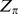 is the size of the lineage descending from 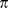 that survived the first round of selection and did not recombine. The dynamics of the lineage size 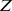 over one population life cycle is described by:
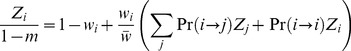
(2)


Eq (2) can be solved for 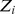 and the solution substituted into (1). The resulting equation makes it possible to express the mean fitness 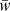, a population characteristic, in terms of parameters that govern the dynamics within a single lineage.

#### Frequencies of the foreign genotypes

We now examine the equilibrium frequency dynamics of the intermediate length genotype 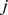. Its frequency 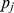 is decreased due to migration, selection and recombination into genotypes with fewer immigrant alleles, but increased due to recombination between genotypes with a greater number of immigrant alleles and the resident genotype [17]. The frequency of the initial immigrant genotype 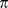 can only be increased by migration at rate *m*. This leads to the following simple recursions:
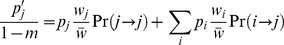
(3)for any genotype other than 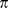, and
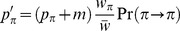
(4)for the initial immigrant type. At equilibrium, the corresponding frequencies 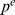 are:
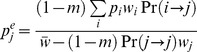
(5)and



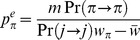
(6)One can see that Eq. (5) and (6), which completely describe the genetic structure of the population at migration-selection balance, contain only those parameters that determine the branching process within a single lineage, except for the mean population fitness 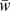. It, in turn, can also be derived from the single lineage dynamics using Eq. (1). To obtain further results, we will make some simplifications of the probability function 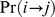 that maps the transition between genotypes, and consider a specific model of constructing the fitness 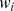 of the foreign genotype.

### A Specific Model: Introgression of Linear Blocks

#### Multiple crossovers on a linear chromosome

The need to follow the frequencies of a large number of genotypes, the number of which increases exponentially with the number of loci, is a major obstacle in multilocus population genetics. A popular solution is to lump the genotypes into classes that share the same number of alleles of a certain kind: we adopt this approach here as it is particularly useful in models of gene exchange between populations, where one is typically concerned with just two types of genes (i.e. the resident and the foreign alleles). Following Barton [17], we assume that the foreign genes in the initial immigrant genotype are evenly distributed along a single chromosome, with a recombination rate *r* between the two neighboring genes. This arrangement will be called a continuous *block* of genes [29], [30], [32]. The most important characteristic of the block is its length, i.e. the number *k* of the foreign genes it carries, 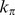 represents the length of the initial immigrant block, 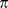. A single crossover on a block will preserve the order and the distance between genes, but if more than one crossover occurs on a block, the actual map distance between the two neighboring foreign genes in the resulting daughter blocks may no longer be uniform. We neglect this issue and treat the daughter genotypes resulting from multiple crossovers as continuous blocks. That is, two genes next to each other will be assumed to recombine with the same probability *r* as two genes separated by any number of the resident loci. We will demonstrate that although this assumption appears difficult to justify biologically, it almost always results in a more accurate estimation of the population parameters than restricting the number of crossovers to one. We therefore only need to keep track on the lengths of the parental and daughter blocks, *k* and *l*, and the probability of transition between full genotypes, 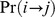, can be replaced by the probability of transition of the block from one length class to another:
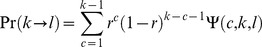
(7)where the function 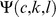 returns the number of daughter blocks of length *l* that could be obtained from all combinations of *c* crossovers on the parental block of length *k*.

#### Fitness function with epistasis

Approximating the multilocus genotypes as continuous gene blocks requires that the fitnesses of individual genotypes must be chosen such so they only depend on the number of foreign alleles and not on their positions. This is a reasonable assumption given a large degree of independence of gene function from the minor changes in its genomic localization. We will parameterize the fitness of a block of length *k* as follows (Nick Barton, pers. comm.):
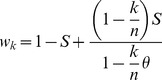
(8)


**Figure 2:**
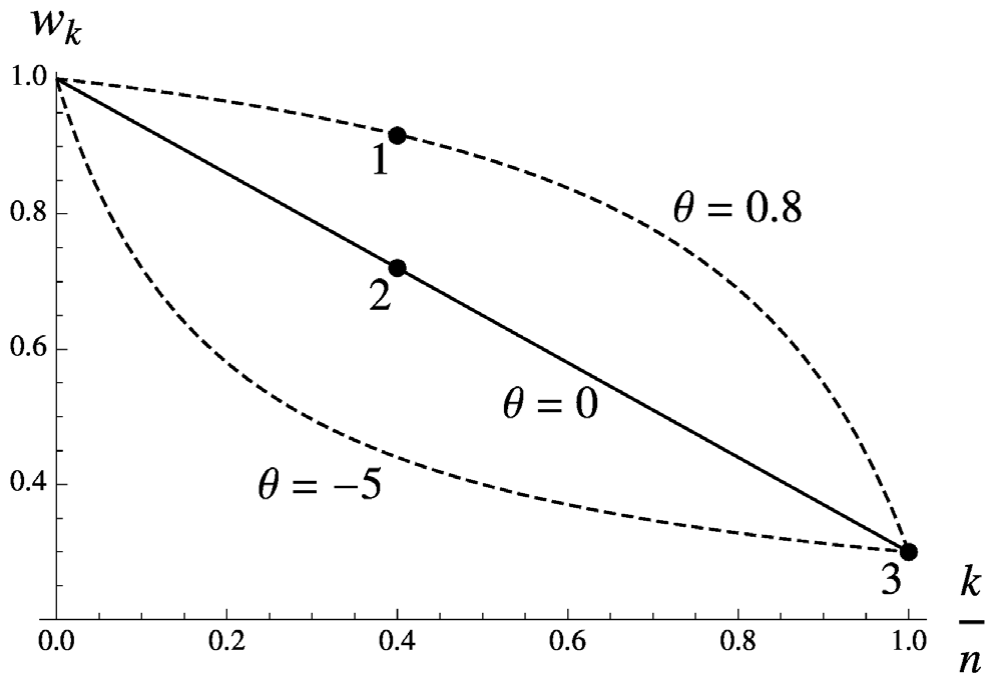
Genotype fitness as a function of the block length. The fitness of the genotype 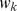 is plotted against the proportion of the chromosome 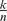 occupied by the foreign genes: 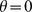 corresponds to additive fitness (no epistasis), while 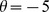 and 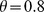 represent the positive and the negative epistasis, correspondingly. The points indicate the fitness of the initial block carrying 4 genes 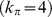 at 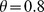. Point 1: *n* = 10, fitness is calculated according to eq. 12. Point 2: *n* = 10, fitness is rescaled by the length of the initial block. Point 3: initial block carries the maximum number of genes, 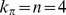.

We use the ratio 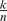, where *n* is the arbitrarily chosen maximum number of loci that contribute to the local adaptation in the resident environment ( = the length on the foreign chromosome in Barton [17]), to establish a lower limit to the fitness of the worst possible genotype, which for 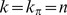 is 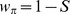, where *S* is the selection acting on *n* genes. At this point, the fitness is independent of the parameter of epistasis *θ*. For any smaller block, 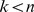, however, the fitness is an increasing function of *θ*, (*θ* <1), and at *θ* = 0, selection acts according to the additive scheme, 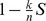 (Fig. 2). When negative, the parameter *θ* also imposes relatively stronger selection on smaller blocks than on larger blocks (positive epistasis). Positive values of *θ* in the feasible range of 0< *θ* <1 impose relatively stronger selection on the smaller blocks than on the larger blocks (negative epistasis, Fig. 2).

Allowing for the initial block to carry only a fraction of the maximum number of selectable genes, 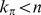, makes it possible to compare the introgression of blocks of different lengths within the same parameter space. This is useful in the case where the populations involved in gene exchange have undergone incomplete divergence (i.e. the hypothetical diverging source population is still climbing the corresponding adaptive peak), as opposed to a scenario 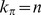 where further divergence is unlikely (the source population has settled on the peak). If *n* is constant and 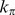 is varied, selection pressure on the initial block increases with its length, whereas if 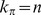, and both are varied, the same selection pressure is distributed among different number of genes, that is, a polygenic trait can be compared to the phenotype controlled by only a few major loci. Unless specified otherwise, the following results were obtained holding *n* constant and varying 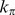, 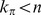.

### Numerical Results: Branching Process and Simulations

**Figure 3:**
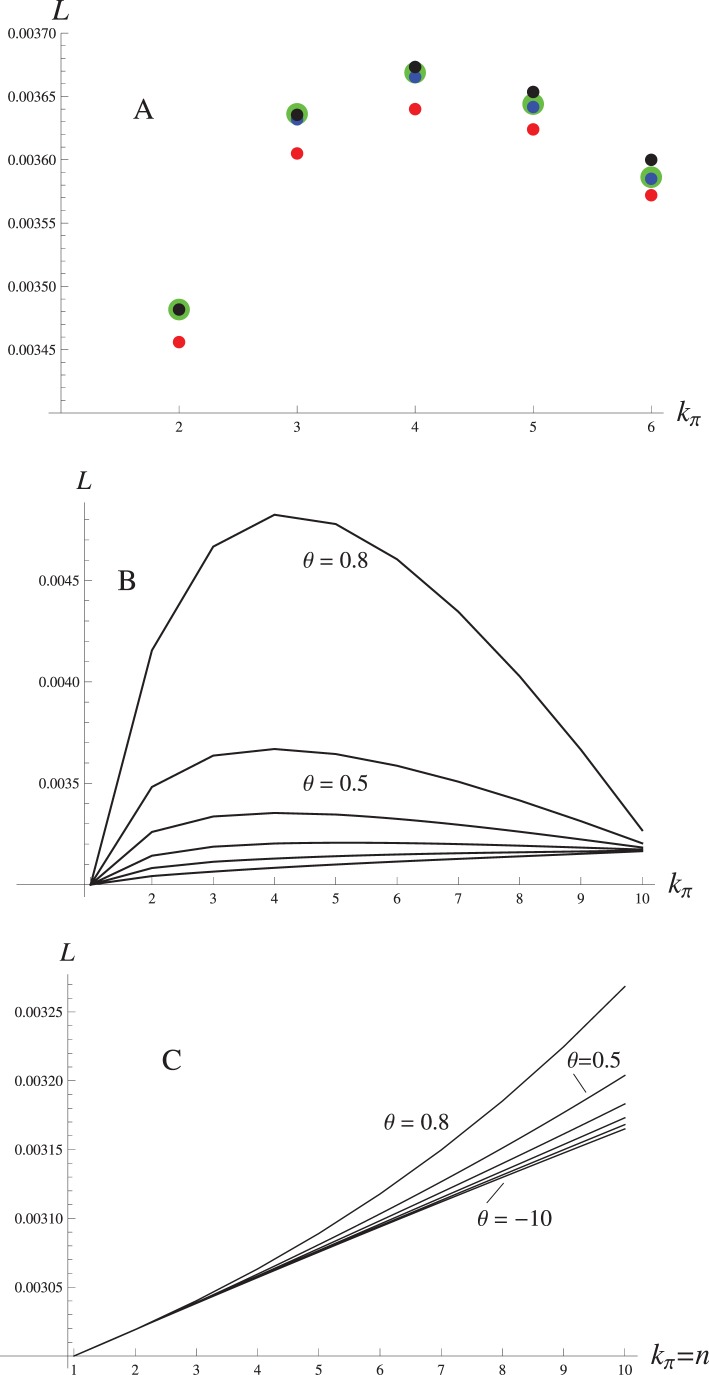
Migration load at the migration-selection balance. **A** – Comparison of the migration load (*L*) imposed by the introgression of 2 to 6 genes in the initial block, calculated from the Branching Process Approximation (BPA, green dots), Panmictic Simulation (PS, red dots), Introgressive Simulation (IS, blue dots) and Single Crossover Simulation (SCS, black dots). Parameter values are: *S = *0.7, *θ* = 0.5, *m* = 0.003, *r* = 0.015, *n* = 10. **B** – Migration load (*L*), calculated from the BPA, as a function of the length (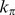) of the initial block. The curves (from bottom to top) correspond to *θ* = −10, −3, −1, 0, 0.5, 0.8. Parameter values are: *m* = 0.003; *r* = 0.015; *S* = 0.7; **C** – the same as in B, but 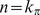.

**Figure 4:**
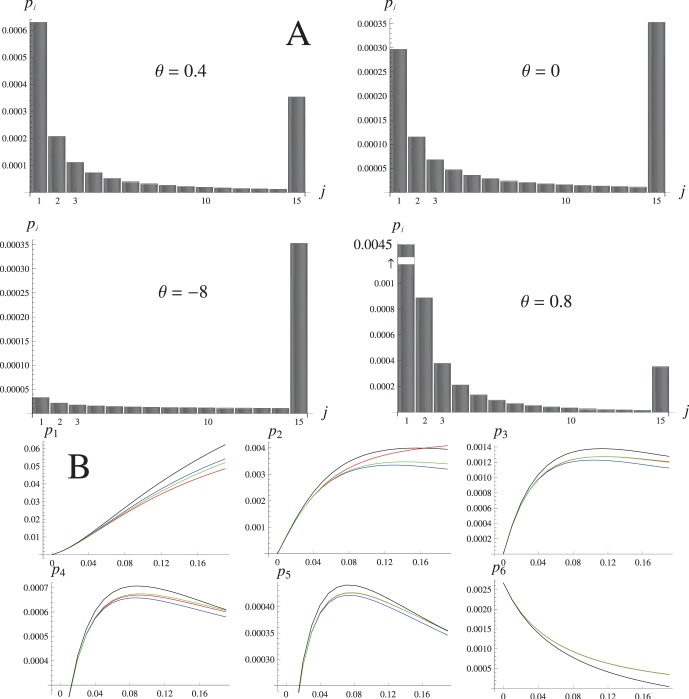
Distribution of the foreign genotype frequencies at the migration-selection equilibrium. **A** – Result of the introgression of the initial block composed of 15 genes, obtained through the Branching Process Approximation (BPA) by solving the Eqs (3) and (8–9) numerically. Note that the actual frequency of the single-gene blocks at *θ* = 0.8 is much higher than the corresponding bar height on the plot (indicated by the larger font on the Y-axis). **B** – Comparison of the frequency distributions, with the initial block composed of 6 genes, obtained through BPA (green line) against Panmictic Simulation (PS, red line), Introgressive Simulation (IS, blue line) and Single Crossover Simulation (SCS, black line) as the recombination rate (*r*) increases. For the simulation results to be comparable with the BPA, different genotypes were grouped together by the number of genes (*j*) they carry. Each plot represents variation in the frequency of the corresponding gene block (1–6). Parameter values are: *S = *0.5, *θ* = 0.2, *m* = 0.001.

The Eqs (1) and (4–5) can be solved numerically to obtain the characteristics of the population at migration-selection equilibrium, termed the Branching Process Approximation (BPA) in the following. Note that the existence of the equilibrium itself depends on the numerical values of the corresponding parameters, and even when the equilibrium exists, the branching process can adequately describe the population dynamics only when the foreign genotypes are rare. While finding the conditions of existence for the equilibria in the multilocus system is outside the scope of our paper, we established the validity of the numerical results by comparing them with the deterministic simulations where some or all of the BPA assumptions were lifted. The times to compute the numerical solutions are of orders of magnitude shorter than those required to perform the corresponding simulations: this allowed for a faster and deeper exploration of the parameter space. We concentrated on low values of the migration (*m<*10^−2^) and recombination (*r <*10^−2^) rates, and medium to strong selection (*S >*0.4), since in this region of parameter space the introgression of the foreign genes is most likely to follow the branching process (Fig. 3A, Fig. 4B). Restricted recombination between loci that contribute to hybrid incompatibility has been inferred among many interbreeding taxa, with most obvious cases often resulting from the chromosomal rearrangement events [33].

When compared to the simulations, the branching process model was found to be a good approximation for the slow introgression at multiple loci [29]. Details are available in the Mathematica notebook (File S1). Note that although Eq (1) for the mean population fitness has *k* roots, corresponding to *k* linear block genotypes, only the largest root is both stable and globally attractive, and was therefore used in the analysis.

#### Extent of introgression and migration load

Consistent with established theory, introgression of gene blocks is limited when selection is strong and recombination rate on a linear block is low. In our model, selection against gene blocks can be further strengthened or weakened by the epistatic interactions. In particular, increasing the parameter of epistasis *θ* facilitated introgression and increased the migration load. We examined the effect of varying the length of the initial block 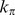 in respect to the fixed maximum number of loci *n*. Introgression of both very small and very large blocks resulted in lighter load, while blocks of intermediate length generally caused the heaviest load (Fig. 3B). This is due to the fact that both small and large blocks have limited opportunity of introgression into the resident gene pool: the small blocks penetrate the selection barrier easily, but can only produce short lineages, while the large blocks are confronted by a very strong barrier at the point when they are introduced into the native population. Since negative epistasis causes even stronger selective pressure on the large blocks, the convex shape of the load on Fig. 3B becomes more pronounced as *θ* increases. Note in Fig. 2, that the full range of epistatic interactions depends on *n* and not on 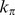, reflecting the assumption that epistasis is determined by the adaptive landscape rather than the degree of divergence between populations. We used the replacements 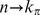 and 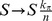 in the Eq (8) to rescale both the epistasis and the strength of selection according to the length of the initial block (point 2 on Fig. 2), but results were qualitatively similar to those presented on Fig. 3B (given in the SI). However, if the same selection pressure *S* was distributed among different number of genes, that is, when the initial and the maximum block lengths were varied as a single parameter 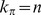, the migration load increased monotonically (Fig. 3C).

#### Distribution of Block Frequencies and the Effective Selection on Individual Genes

Irrespective of the value of the epistasis parameter, the frequency distribution of the block lengths is always bimodal, with the peaks corresponding to the smallest (*k* = 1) and the largest 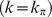 blocks (Fig. 4A). Note that the frequency of the largest (initial) blocks is replenished every generation by migration, and we choose to calculate the block frequencies consistently after migration but before selection, i.e. at the same time when the migration load is measured. While estimating the frequencies after selection would result in a decreased frequency of the initial blocks, it otherwise does not affect our conclusions. There is a gradual transition from the domination of the largest blocks under the strong positive epistasis (*θ* <0) to the domination of the single-gene blocks under the negative epistasis (*θ* >0, see Fig. 4A). For most part of its feasible range, the largest and the smallest block frequencies are of comparable scale: however, as *θ* approaches 1, the frequency of the single-gene blocks increases very rapidly: for example, at (*θ = *0.8, S = 0.7, *r* = 0.01 and *m* = 0.001) *p*_1_ is an order of magnitude higher than *p*_15_ (Fig. 4A). To demonstrate the extent of introgression by a single variable, we calculated the effective selection pressure on the individual genes, s* [17]. This is simply the ratio of the migration rate, *m*, to the average weighted frequency of the single alleles at equilibrium, 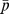:
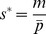
(9)where
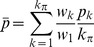
(10)


**Figure 5:**
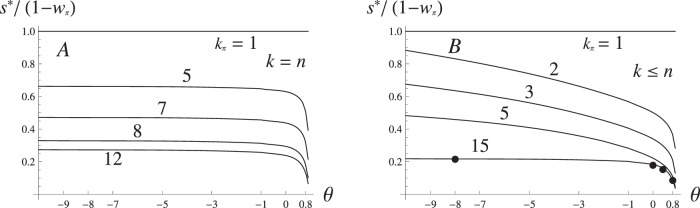
Effective selection (s*) on the individual genes. **A** – s* as a proportion of the total selection on the initial block, with the number of genes in the initial block varying from 1 to 15, and epistasis *θ* ranging from −9 to 0.8. The maximum number of genes is fixed at *n* = 15. **B** – the same as in B, but the maximum number of genes, *n*, is set to equal 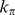
in each case. Points along the curve for 15 loci correspond to the frequency distribution plots on A. Other parameters: *S* = 0.7, *r* = 0.01, *m* = 0.001.

Note that 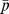 here accounts for the non-additive selection on the individual alleles, whereas in Barton [17] only the additive fitness 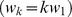 was considered. The effective selection pressure roughly shows how much of the foreign genetic material is present in the resident population, relative to the number of migrants that arrive every generation. It takes the maximum value of 1 if there is no introgression (i.e. with positive migration rate, *m*, the fitness of the initial block, 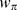, must be 0) and drops down to *m* if gene flow swamps the resident population. We found that the effect of epistasis on the effective selection pressure is different from that on the distribution of block frequencies above. For the most part of the feasible range of *θ*, s* stays almost constant, but then drops abruptly when epistasis becomes strongly negative (*θ* >>0): this corresponds to the rapid increase in the frequency of the single gene blocks in the same parameter range (Fig. 4A, Fig. 5A). The change in s*, however, is much more gradual when the size of the initial block is less than the maximum allowed block length 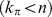, Fig. 5B).

### Invasion of the Modifiers

Since we are ignoring the gene positions on a linear block, it is not possible to estimate the strength of selection acting on the individual foreign genes. It is feasible, however, to follow the fate of the marker gene *x* located at a considerable distance from the foreign block, such as that the recombination rate between the marker and the edge of the linear block, 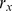, is much larger than the rate of recombination within the block itself 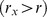. The barrier to the gene flow at the neutral marker linked to a block of genes under selection has been calculated by Barton and Bengtsson [18], by considering a matrix of all possible foreign backgrounds which the marker can be associated with. We use the same method here, but assume that the marker is a novel mutation equally likely to originate on both the foreign and the resident backgrounds. Instead of focusing on the barrier to gene flow, we investigate the fate of the mutation that reduces, to a certain degree, the deleterious effect of the foreign genes in a resident environment. In the fitness formulation used here, such mutation (modifier) can alter two key parameters in the Eq. (8): the strength of selection (*S*) and epistasis (*θ*), by the corresponding replacements 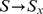 and 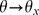. We assume that the modifier’s effect is manifested at the diploid stage, and since the foreign genes are found only in heterozygotes, the mutation is analogous to the modifier of dominance [34]. Note that in case the dominance at several loci needs to be modified at once: in principle, this is possible in polygenic systems with a few genes responsible for most of the variation in a trait and the remaining variation being affected by a much larger number of loci. For example, the distinctive wing coloration patterns in a butterfly *Heliconius erato* are controlled by three loci of major effect, expressed early in the wing development, while multiple QTLs shape the minor details during the later stages of development [35]. A mutation targeting genes early in the pathway would therefore interact with multiple genes expressed later on. Alternatively, the modifier can affect the recombination rate on its background genotype by the replacement 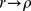, without changing the fitness of the foreign genotype [36]. It follows from Eqs (5–6) that reduction of the recombination rate always limits the introgression of the foreign block into the resident population, thus improving the population mean fitness. The following recursions describe dynamics of the frequency 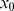 of the modifier on the resident background:
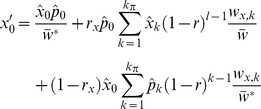
(11)and the frequency 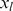 of the modifier associated with the foreign block of length *l* :



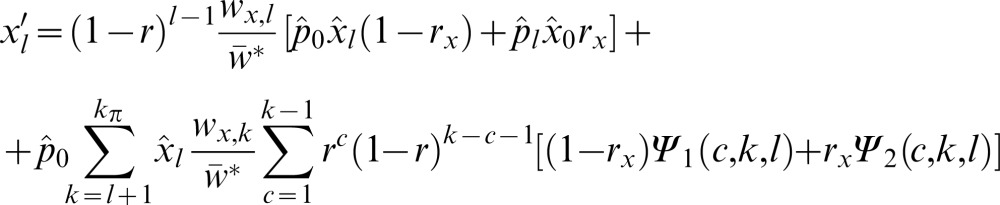
(12)Here, the «hat» superscript indicates the frequency of the corresponding type after migration took place, e.g. 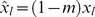 for all 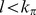 and 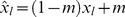 for 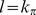; 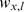 is the fitness of the foreign block associated with the modifier of selection or epistasis. For the first time, we consider the frequency 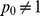 of the resident gametes, calculated simply as 
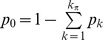
. Since the modifier effect is manifested in diploids, the mean population fitness has to be estimated after the unions of gametes, and is calculated as:
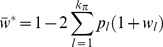
(13)


In the case where modifier only alters the recombination rate, the fitness of the block remains unchanged: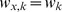, 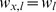, but the background recombination rate *r* is replaced by *ρ* in the presence of the modifier. Differentiating the system (11–12) in respect to 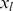 results in a matrix of linear coefficients: the leading eigenvalue of this matrix, *λ*, represents the strength of selection acting on the modifier [20] while the population is still in the migration-selection equilibrium. As follows from the Eqs (11–12), the dynamics of the invading modifier is determined both by its own effect on the background genotype and by the population structure/parameters at the migration-selection equilibrium.

#### Modifiers of selection, epistasis and recombination in a common parameter space

We systematically explored the parameter space in the Eqs (5–6) and (11–12) and numerically estimated the leading eigenvalue of the invasion matrix *λ*. Note that in our parameterization, the selective advantage due to the modifier mutation cannot exceed the fitness of the resident genotype: the modifier invasion rate *λ* is therefore bounded by the difference between the (background) parameters of the migration-selection balance (i.e. *S*, *θ*, *r*) and their corresponding altered values in the presence of the modifier 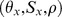. That is, the favorable conditions for the invasion of selection, epistasis and recombination modifiers are 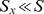, 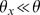 and 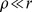, respectively. The effects of background parameters on the invasion rates of all three modifier types are complex: we investigate these to some extent in the SI. In the following, we concentrate on the effect of the modifier’s own properties, and arbitrary choose common set of background conditions that allow for the comparison between the different modifier types.

**Figure 6:**
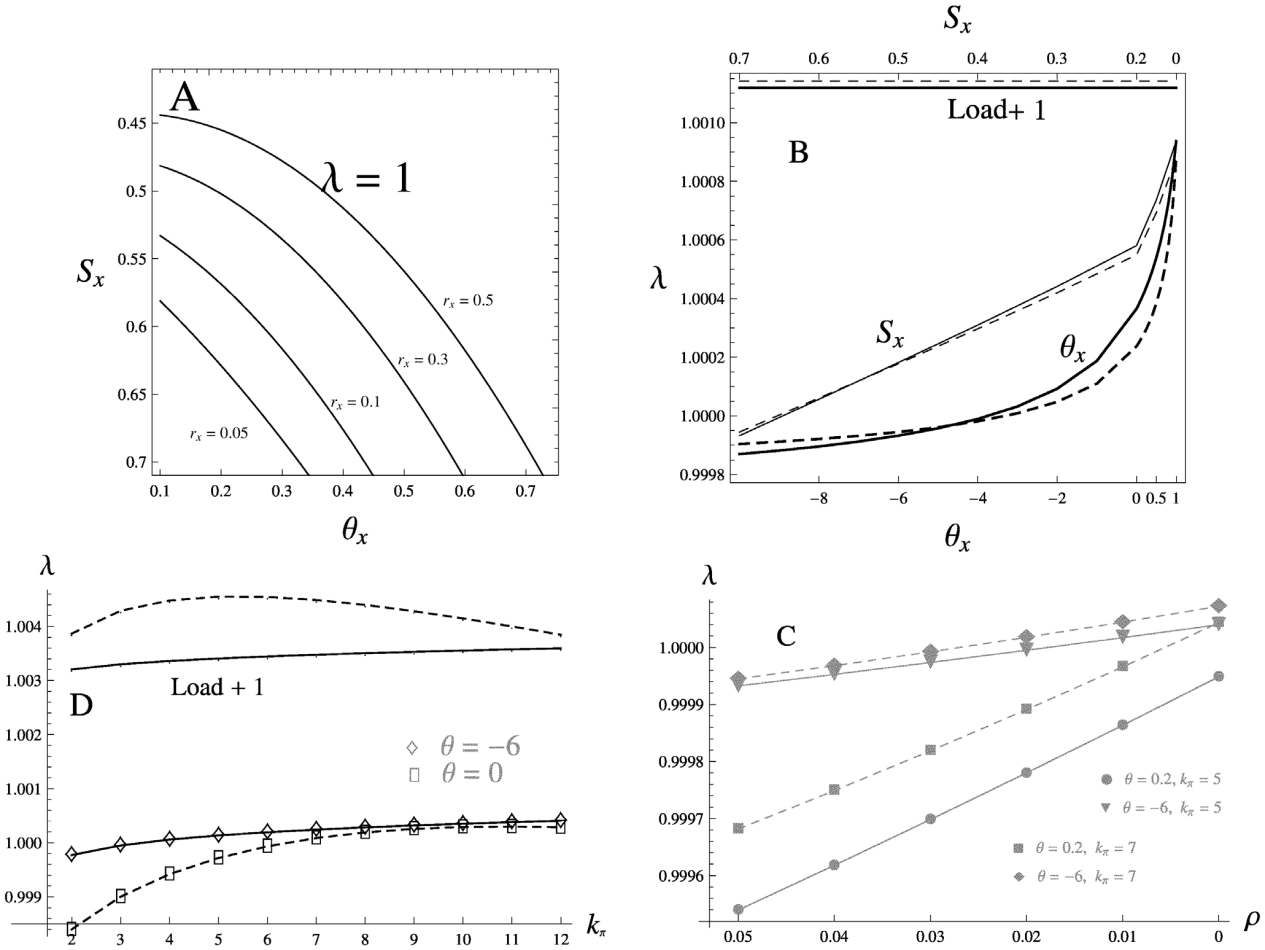
Selection acting on the invading modifier. **A** – view of the parameter space with the modifier selection strength 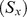 and epistasis 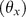 plotted on the X and Y axes, the leading eigenvalue of the invasion matrix (λ) equals one along the lines shown for the following values of 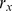
(top to bottom): 0.5, 0.3, 0.1, 0.05. The modifier is able to invade in the space above each line, and cannot invade below the line(s). Other parameters: *S* = 0.7, *θ* = 0.2, *m* = 0.003, *r* = 0.001, *k_π_* = 5. **B** – λ as a function of the modifier epistasis, 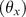, plotted on the bottom axis; and selection strength 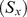, plotted on the top axis. The migration load (which is independent of 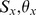) is shown by the horizontal line on the top. Solid lines indicate *k_π_* = 5, dotted lines indicate *k_π_* = 7. Other parameters: *S* = 0.7, *θ* = –6, *m* = 0.001, *r* = 0.05, *r_x_ = *0.1, *n* = 10. **C** – Invasion rate of the modifier of recombination, as a function of the imposed recombination rate *ρ*. Markers indicate different values of the background epistasis, solid lines: *k_π_* = 5, dotted lines: *k_π_* = 7. Other parameters are as in B. **D** – Modifier of recombination invades non-monotonically with the length of the initial block, *k_π_*. Solid lines: *k_π_* = 5, dotted lines: *k_π_* = 7; markers indicate different values of the background epistasis. Migration load is shown at the top of the plot. Other parameters: *S* = 0.7, *m* = 0.003, *r* = 0.05, *r_x_ = *0.1, *n* = 12.

In general, the modifier of selection and epistasis are able to invade with the comparable rates under a wide range of conditions. The parametric plot on the Figure 6A shows that increasing the selection and epistasis differentials have similar effects on the invasion threshold *λ* = 1: the space where invasion is possible gets narrower as the linkage between the modifier and the foreign gene block (*r_x_*) becomes loose (Fig. 6A). Even an unlinked modifier 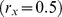 that simultaneously confers large selective advantage and switches the epistasis from positive to negative can invade the resident population. Reaching the invasion threshold for the unlinked modifier of recombination, however, requires much more stringent conditions, most of all a very high background recombination rate (*r* ≈ 0.2). At these values of *r*, our model based on the branching process is no longer a good approximation to the multilocus introgression (Fig. 3A, 4B): we therefore assumed a tighter linkage to the foreign block (*r_x_* = 0.1) to ensure that invasion is possible in the following examples. Throughout the parameter space, the invasion rate *λ* is an exponentially increasing function of the altered epistasis parameter, 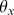, and a linear function of the modifier selection parameter, 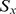. In the example shown on Fig. 6B, the maximum strength of selection acting on the modifier approaches (but can never exceed) the migration load. The background parameter set on Fig. 6B intersects with that on Fig. 6C, where *λ* is plotted as a function of the altered recombination rate *ρ*. Although comparable, the invasion rate of the recombination modifier is much less than that of selection/epistasis, and the threshold for invasion is only reached if the recombination on a block is heavily suppressed or completely arrested (*ρ* ≈ 0). Note that due to our model being a good approximation only when the linkage between foreign loci is sufficiently tight, we could not compare the case where the modifier of recombination is expected to spread with the fastest rate, i.e. where the recombination between the unlinked immigrant genes is arrested by the modifier. To find out if the modifier arresting recombination (from the value of *r* = 0.05) on the larger blocks is favored over that on the smaller blocks, we set *ρ* = 0 and estimated *λ* against *k_π_* under the varying background conditions. The invasion rate was indeed increasing with the initial block length, though still being of order of magnitude smaller than the corresponding migration load (Fig. 6D). Under the negative epistasis (*θ* >0) the modifier arresting recombination on very large blocks (*k_π_* >10) invades marginally slower than that on the blocks of moderate length: this parallels reduction in migration load imposed by large initial blocks (Fig. 3, Fig. 6D).

## Discussion

In this article, we approximated introgression at multiple loci based on the assumption that the foreign genotypes occupy a very small proportion of the resident population and that the migration-selection process is at equilibrium. First, a general analytical description of introgression as a multi-type branching process was derived, based on the transition probability between recombining genotypes; we then simplified it further by assuming a specific model of gene blocks, where the population mean fitness and the genotype frequencies could be found numerically. Finally, we demonstrated how these equilibrium results could be used to investigate the invasion of a modifier mutation that masks the deleterious effect of gene flow, or suppresses recombination between the immigrant genes. Bearing in mind that our model only provides a good approximation at the low migration, low recombination scenario, we found wide parameter ranges where selection for the masking modifier was significant relative to the migration load imposed on the resident population, but narrower range of feasible conditions allowed for invasion of the recombination modifier.

### Comparison to Barton (1983)

Modeling introgression as a branching process where the linear gene blocks, explicitly characterized by the number of genes they carry, are being broken by recombination goes back to Barton [17]. He treated the resident population as (i) an unlimited pool of mating partners, which (ii) has essentially no effect on the dynamics of branching lineages. In the present paper, we kept the first assumption, but lifted the second one: that is, the foreign genotypes were still allowed to mate freely, and exclusively, with the residents, but selection against the migrants was normalized by the mean fitness of the resident population, 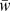. While Barton’s study mostly concerned the balance between selection and recombination rates, which ultimately determined whether introgression is limited or indefinite, we concentrated on the effect of the (limited) introgression on the resident population structure. Unless the immigrant lineages can be treated as independent, valid approximation of this effect by the branching process is not possible: this is why our numerical results are presented for such parameter ranges (low migration and recombination rates, and strong selection) that keep the total fraction of the introgressed genetic material low at equilibrium. Other distinctive feature of our model is that multiple crossovers per gene block are allowed, rather then just a single crossover [17], [29]. While our approach leads to correct (binomial-like) distribution of the daughter block lengths following recombination of the initial immigrant block of 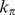 genes, it underestimates the recombination rate at the discontinuous daughter blocks that contain «gaps» between the foreign genes. The single crossover model, however, gives an incorrect (uniform) distribution of the daughter block lengths, and ignores the discontinuous blocks altogether. The position-independent model, and the single crossover model, respectively, under- and overestimate the frequencies of the small blocks, but the differences between the two models are small as long as the recombination rates are low (Fig. 3A, 4B).

In our model, selection on different genotypes within the immigrant lineage depended significantly on the epistatic interations within a gene block. Yet, the effect of introgression estimated on the population level (migration load, Fig. 3) and on the level of individual genes (the effective selection pressure s*, Fig. 5A,B), revealed that epistasis only has a strong overall effect if the deviation from additive fitness extends to the initial immigrant genotype. If, however, the initial genotype is insensitive to epistasis, as in the case of 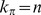, the consequences of fitness non-additivity are much more subtle, and are visible primarily on the shape of the block frequency distribution (Fig. 4A). This is because the smaller blocks represented at higher frequency under the negative epistasis experience relatively weaker selection than they do under the posisitive epistasis, despite being less abundant in the latter case. Our results thus suggest that under a specific assumption that epistasis is manifested in the offspring of the initial block but has little of no effect on the initial block itself, the same amount of load and per-gene effective selection can be generated with very different observable patterns of introgression.

### Alternative Microevolutionary Processes at the Migration-selection Balance

In the presence of the maladaptive gene flow, selection should favor those resident genotypes that have less chance of being associated with the immigrant genes. An allele that contributes to a stronger reproductive barrier between the residents and the migrants, through genetic, ecological, behavioral or other incompatibility, should therefore invade: current theory and empirical evidence suggest that that reinforcement or prezygotic isolation can occur in many systems [10], [37], though it is not particularly likely in a one-way migration scenario [9]. Alternatively, a rare variant, which improves fitness while being found with the immigrant allele, relative to the most widespread type in the population, can also be favored: whether this mechanism operates in nature remains to be demonstrated, but models of the evolution of dominance in spatially heterogeneous environment [21], [25] and invasion of gene duplication in response to gene flow [26] suggest that it is at least likely under a wide range of conditions. Moreover, an analysis of fitness gradients for mate choice versus the modification of dominance [38] suggested that the latter is more strongly selected when allele frequencies at the single locus with heterozygote disadvantage are unequal: the situation where rare migrants are introduced into a large resident population satisfies this criterion. Here, we showed that robustness to the deleterious effect of immigrant alleles in a multi-locus system can evolve at the migration-selection balance, but the question of whether this scenario poses a likely alternative to the reinforcement of the reproductive barriers remains open. A direct comparison of selection strengths acting on the modifier(s) of assortment [22] with the modifiers of selection/dominance/recombination is therefore needed: a study on this subject is currently under way.

### Evolution of Recombination Rate

In a multilocus system, a strong barrier to gene flow exists even in the absence of any mechanisms of pre-mating isolation [18]. This is due to the fact that recombination is generally a much slower process than selective elimination of individuals carrying the maladaptive foreign alleles in linkage disequilibrium: physically reducing the rate of recombination between the foreign genes would therefore make the barrier even stronger. A number of models of migration-selection balance suggest that suppression of recombination/clustering of the locally adaptive loci in the genome is favored by selection [27], [28], [39], [40]. Here, we have demonstrated that tightening linkage between the immigrant genes will help to eliminate them from the population early in the lineage, thus decreasing the proportion of the population that carries any deleterious material. The modifier that reduces the recombination rate will have a relatively higher chance to be preserved on the resident background and therefore is favored by selection (Fig. 6C,D). However, under the premise that the linkage on the introgressing block is already tight, further reduction, or even complete arrest of recombination cannot be selected too strongly. This caveat could explain why a direct comparison between the invasion rates of the modifiers of selection/epistasis and the modifier of recombination revealed much weaker selection for the latter (Fig. 6B,C).

### Effect of the Initial Block Length

Despite the fact that, after recombining into the resident gene pool, the foreign blocks of different length are selected independently at any single time point, selection on the larger blocks negatively affects the smaller blocks in a descending lineage. Increasing the number of genes in the initial block has therefore a dual effect on the lineage size: even though maladapted genes carried by large chunks of the genome are inherited by a larger number of descendants, elimination of the large blocks early in the lineage can be so effective that their impact on the equilibrium population fitness, measured as the migration load, becomes less relative to that caused by the introgression of the blocks carrying moderate number of genes (Fig. 3). This non-monotonic effect on the migration load is also manifested on the invasion rates of various modifier types, though with much weaker intensity (Fig. 6D). While a modifier mutation rescuing a (single) large block has a stronger selective advantage, it can still have less chance of being associated with the foreign alleles if the introgression is limited by the initial block size. Note that the standard matrix method of calculating the invasion rate employed here does not distinguish between the modifier originating on any specific genetic background. An alternative method, and the only feasible approach of examining the modifier that is tightly linked to the foreign block, would be to calculate the speed of the branching process starting from the modifier mutation associated *with the particular genotype*, and verify the results against simulations: we are currently working on this. So far, our results only seem to suggest that the gene flow dynamics in heterogeneous populations may depend non-linearly on the size of the genomic region containing the locally adapted loci.

The recent advance of population genomics, with large datasets becoming available for a number of hybridizing taxa, allows for an empirical application of the theoretical results such as [17], [29], [30] and ours. In particular, the introgression of genomic blocks on the flanks of the hybrid zone between the eastern and western subspecies of the house mouse (*Mus musculus musculus* and *Mus m. domesticus*) in Central Europe has now been traced in unprecedented detail [41]. Modeling this process based on the existing block size data is currently under way.

## Materials and Methods

### Derivation of Eq (1) for Mean Population Fitness

**Figure 7:**
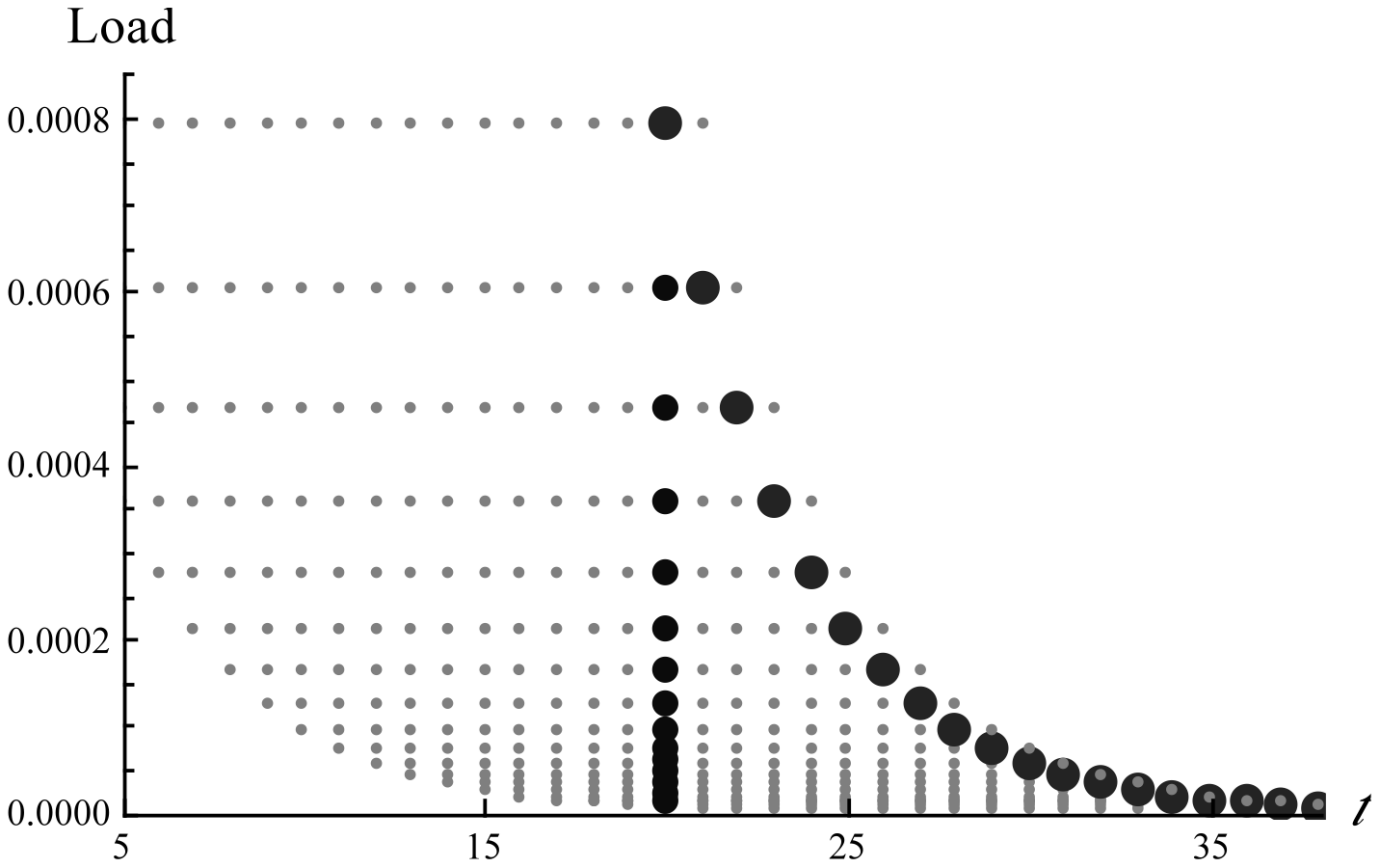
Migration load at the migration-selection balance. The reduction in mean population fitness caused by the descendants of the migrants arrived in the generation 20 is measured every generation that follows (large black dots); this is equivalent to the load caused by the progeny of the migrants arrived in all preceding generations, calculated at point *t* = 20 (smaller black dots). The mean population fitness at any time can therefore be calculated from the branching process within a single lineage, according to Eq (1).

Let us examine a lineage 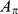 descending from a single initial immigrant genotype 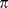 introduced at the generation 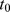, and assume for simplicity that exactly one immigrant arrives every generation. A subset 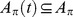 will represent all members of the lineage present after migration and before selection, at each of the successive generations following the introduction: 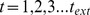, where 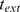 is the generation when the last member of the lineage becomes deterministically extinct. The ultimate extinction of the lineage is guaranteed by our previous assumption that recombination cannot break up the foreign genotypes beyond a single gene; once this limit has been reached; the single gene is guaranteed to eventually be eliminated by selection [42]–[44]. Because the number of generations after which the lineage is sampled, *t*, is counted relative to the time of introduction 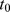, and the migration-selection process is at equilibrium, varying the time of introduction will have the same effect as varying the time of sampling. It is easy to see that in our model, where no interaction between the foreign genotypes is assumed, population at any time can be represented as a set of *independent* lineages with different times of origin, but otherwise identical (Fig. 7). Moreover, since the initial immigrant genotype is introduced at the same rate every generation, the set 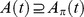 of all lineages sampled at time *t* will be equivalent to the set 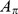 representing a single lineage:
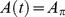
(14)


Eq (14) will hold for any number of immigrants arriving each generation, as long as the migration rate *m* is constant. The left-hand side of (14) can be re-written in the frequency representation, as a sum 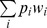, where 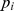 is the frequency of the genotype *i* in the current generation after migration but before selection and 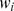 is genotype fitness. Adding the fraction of the resident genotype after migration, 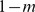, we obtain the expression for the mean population fitness, 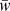:
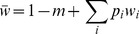
(15)


**Figure 8:**
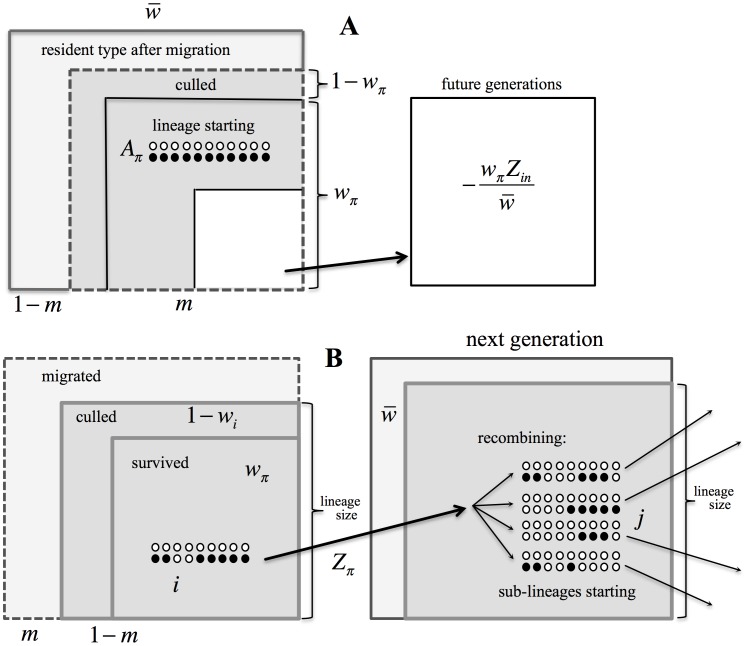
Schematic representation of the terms in Eqs (1) and (2). **A** – representing the mean population fitness from the branching process within foreign lineages. The future load caused by the independent lineages (white contour) is subtracted from the fitness contribution of the migrants, measured at the generation of sampling (contour filled by dark grey). Note that the proportion of migrants culled before reproduction, 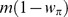, is not a part of the mean population fitness. **B** – Dynamics of the lineage size, 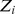, determined by the Eq. (2). 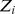 is reduced by migration and selection, but increased as the foreign genotype recombines and gives rise to sub-lineages. Note that the term 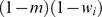, i.e. the contribution of the individuals culled by selection, is included in the lineage size calculation.

We must now re-write the right-hand side of (15) in terms of the relative frequencies of all the genotypes within a lineage, sampled at different time points. It will include the frequency of the initial genotype 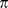 that immigrated and have been selected against in the current generation, 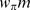, minus the fraction of the population mean fitness, 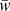, occupied by the progeny of 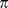 destined to become extinct in the future (Fig. 8A), resulting in the Eq (1).

### Derivation of Eq (2) for the Lineage Size *Z*

The concept of the lineage size *Z* is similar to Haldane’s [44] concept of the number of selective deaths necessary to eliminate a deleterious variant from the population. We calculate *Z* by adding the contributions made by the lineage members through time, and account for the effects of migration, selection, and recombination at each of the consequent generations. The factor 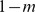 in the right-hand side of the recursive Eq (15) accounts for migration, invariably reducing the lineage size throughout its lifespan. The denominator 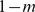 represents the fraction culled in the present generation, while the last term in the right-hand side of (15) is a product of the fraction 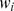 that survives and enters the next generation (hence divided by 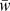) and the sum of sizes of the daughter lineages descending from 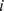 (Fig. 8B). While the explicit knowledge of the transition probability 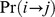 is required to obtain the numerical value of *Z*, in a degenerative case where there is no recombination within the immigrant genotype, 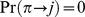, for all *j*, the lineage size can be found exactly:
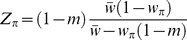
(16)and the only stable solution for the mean population fitness is 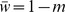, which is a standard result for one-locus migration-selection balance model [45].

#### Derivation of Eq 7 for the transition probability in the linear block model

Let us assume, for a start, that there can only be one crossing-over per continuous block of foreign genes [17], [29]. In this case, the offspring genotypes will also be continuous blocks, and will only differ from the parental block by the number *k* of the foreign alleles they inherit. The probability of getting an offspring block of any size in this case is simply 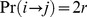 [17]. When multiple independent crossovers are allowed, the probability of recombination occurring at *c* locations chosen from 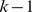 intervals between *k* genes is 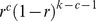, and a number of ways to obtain the daughter block of length *l* is determined by the binomial function 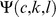:
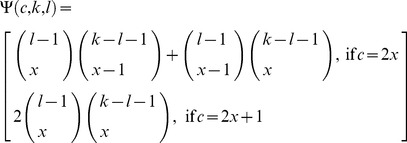
(17)


We derive 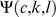 explicitly in the SI. Note that while there is exactly 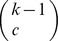 ways of choosing, with equal probability 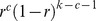, the locations for *c* crossovers among 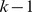 intervals between *k* genes, only one pair of daughter blocks can result from the partition of a single parental block. This means that the function 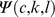 should also be divided on 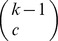, and the corresponding binomial cancels out in Eq (7).

### Simulations Used to Verify the Branching Process Approximation

We verified the validity of Branching Process Approximation against the deterministic, frequency-based simulations of the migration-selection process as it was described in the previous sections. The initial population consisted of the resident genotypes only; the foreign genotypes, represented as identical linear blocks of equally spaced genes, were introduced every generation at the rate *m*, followed by selection and reproduction. The simulations were run using the Multilocus package [46] in *Mathematica* until the equilibrium was reached, at which point the stable genotype frequencies were compared with the corresponding results of BPA. Three models were used. In the Introgressive Simulation (IS), the foreign genotypes could only mate with the resident genotypes, assuming that there an unlimited supply of the residents. At the stage of selection, however, the frequency of the resident genotype was calculated as 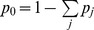, and the mean population fitness as 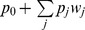. The IS therefore exactly follows Eqs (1) and (3), which describe a general branching process, without the simplifying assumption of all foreign genotypes are represented as continuous gene blocks. If the initial genotype consists of only one (*k_π_* = 1) or two (*k_π_* = 2) genes, the results of the introgressive simulation match the BPA precisely (Fig. 3A, Fig. 4B), but starting from *k_π_* = 3, the IS includes genotypes that carry the same number of genes located at different positions. The deviation of the IS from BPA increases as the number of genes, migration and recombination rates increase. As a modification of the IS, we included the case where the number of crossovers per block was restricted to one, referred to as the Single Crossover Simulation (SCS) in the following. The probability of recombination on a block of length *k* was calculated in SCS as (*k* –1)*r*, which corresponds exactly to the recombination model used by Barton [17].

In the Panmictic Simulation (PS), all mating types, including those between foreign genotypes, were allowed, and the positions of individual genes were recorded in all genotypes. The PS is the most realistic way to describe the population at the migration-selection balance, following the frequencies of 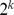 genotypes composed of two (the resident and the foreign) alleles at *k* loci. Selection in PS was acting on haploids, consistent with the selection regime in BPA and IS. Since matings between the foreign genotypes were still very rare, a switch to diploid selection (acting differently on zygotes that carry foreign genes on both homologous sets) had very little effect on the results. As expected, the PS shows larger deviation from BPA than the Introgressive Simulation, with the differences between PS and IS typically being greater than between IS and BPA (Fig. 3A, 4B). The SCS, however, consistently deviated most from the PS in comparison to the IS and BPA. Nevertheless, the deviation remains small in the parameter range where our numerical results were obtained (Fig. 3A, 4B), suggesting that the branching process model is a good approximation for the introgression at multiple loci [29].

### Derivation of the Eq for the Mean Population Fitness at the Diploid Stage

In order to estimate the leading eigenvalue of the modifier’s invasion matrix, we need to calculate the frequency of the resident type, as 
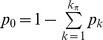
. This does not affect the frequencies of the foreign gene blocks, since we still assume that the foreign genotypes do not compete which each other for mating with the resident type. The mean population fitness 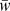 is thus calculated as the frequency of heterozygotes formed by the foreign gametes after selection, 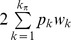, plus the residual fraction of the resident gamete pool that remains after all heterozygotes have been formed, 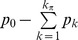. This simplifies to Eq (13).

## Supporting Information

File S1 **A *Mathematica* notebook containing the key functions used to derive the equations and the graphical results in the article.**
(PDF)
