# Supplementary material for: Migration-Selection Balance at Multiple Loci and Selection on Dominance and Recombination
Source: PLoS One. 2014 Feb 14;9(2):e88651. doi: 10.1371/journal.pone.0088651 (PMC3925130; doi:10.1371/journal.pone.0088651)
Supplement: File S1 — A Mathematica notebook containing the key functions used to derive the equations and the graphical results in the article. (PDF) [file pone.0088651.s001.pdf]

Supporting Information I

Analytical

Fitness of the linear block

Fitness of block of length *i*

$$W[i\_] := 1 - S + \frac{\left(1 - \frac{i}{n}\right) S}{1 - \frac{i \theta}{n}}$$

Fitness of the block with a modifier mutation present

$$w[i\_] := 1 - s + \frac{\left(1 - \frac{i}{n}\right) s}{1 - \frac{i q}{n}}$$

Mean population fitness when the frequency of residents is not 1 (used to estimate the amount of selection acting on the modifier)

```
Wbar[k_] :=
 2 (Sum[p[i] W[i], {i, k - 1}] + (p[k]) W[k]) + (p[0] - (Sum[p[i], {i, k - 1}] + (p[k])))
```

Recombination

Derivation of the opeator Ψ in the paper:

We need to find the number Ψ [*c,k,i*] of the daughter blocks of length $i \in [1, k-1]$ resulting from all possible combinations of $c \in [1, k-1]$ crossovers in k - 1 locations. The cases of no recombination (c = 0) and $i = k$ are not included.

First, recall that any one combination of crossovers divides a single parental block in two groups of segments, which then are joined to form the two daughter blocks: we number these as (1) and (2). Notice that when a block of length k is cut by the odd number of crossovers ($c = 2x+1$), the number of resulting segments forming either (1) or (2) is equal $x + 1$. With even number of crossovers ($c = 2x$), the first arbitrary chosen daughter block (1) will be composed of x+1 segments, and the second block (2) will contain x segments.

$x = 1$	(1)	----		----	$x + 1$
$c = 2x + 1$	(2)		-----	-----	$x + 1$
$x = 1$	(1)	----		---------	$x + 1$
$c = 2x$	(2)		-----		x

Consider the case when the two daughter blocks (1) and (2) have lengths i and $k - i$. Let's find out how many ways there are to compose the daughter block (1) of length i with $x + 1$ segments, in both cases of $c = 2x + 1$ and $c = 2x$. This is the same as finding the number of *compositions* of the integer a into b parts, which is $\binom{a-1}{b-1}$. Hence, there are $\binom{i-1}{x}$ compositions of (1). Similarly, we find out the number of compositions of (2): if it contains $x + 1$ segments (i.e. $c = 2x + 1$), there

are $\binom{k-i-1}{x}$ compositions, and with *x* segments (i.e. *c* = 2*x*), there are $\binom{k-i-1}{x-1}$ compositions. To get the total number of compositions of the parental block, the corresponding binomilas need to be multiplied, i.e. $\binom{i-1}{x}\binom{k-i-1}{x}$ and $\binom{i-1}{x}\binom{k-i-1}{x-1}$ for the odd and even numbers of crossovers, respectivelly.

Now, consider the reverse case where the block (1) has length *k* - 1 and the block (2) has length *i.* Then, if *c* = 2*x* +1, the block (1) has $\binom{k-i-1}{x}$ and the block (2) has $\binom{i-1}{x}$ compositions; and if if *c* = 2*x*, the block (1) has $\binom{k-i-1}{x}$ and the block (2) has $\binom{i-1}{x-1}$ compositions.

Combining all these terms to get the total number of compositions of the parental block, the result follows:

If *c* = 2*x*+1*,* then $\Psi[c,k,i] = \binom{i-1}{x}\binom{k-i-1}{x}+\binom{i-1}{x}\binom{k-i-1}{x}$

If *c* = 2*x,* then $\Psi[c,k,i] = \binom{i-1}{x}\binom{k-i-1}{x-1}+\binom{k-i-1}{x}\binom{i-1}{x-1}$

In *Mathematica* code:

```
Y[x_, k_, i_] := If[EvenQ[x], Binomial[i - 1, x / 2] Binomial[k - i - 1, x/2 - 1] +
    Binomial[i - 1, x/2 - 1] Binomial[k - i - 1, x / 2],
   2 Binomial[i - 1, (x - 1) / 2] Binomial[k - i - 1, (x - 1) / 2]]
```

Φ does the same, manually (up to 12 genes below).

```
Ψ[x_, k_] := {Transpose[
    {#[[1]] & /@ Subsets[Range[k - 1], 1], k - #[[1]] & /@ Subsets[Range[k - 1], 1]}],
   Transpose[{#[[1]] + k - #[[2]] & /@ Subsets[Range[k - 1], 2],
     #[[2]] - #[[1]] & /@ Subsets[Range[k - 1], 2]}],
   Transpose[{#[[1]] + #[[3]] - #[[2]] & /@ Subsets[Range[k - 1], 3],
     #[[2]] - #[[1]] + k - #[[3]] & /@ Subsets[Range[k - 1], 3]}],
   Transpose[{#[[1]] + #[[3]] - #[[2]] + k - #[[4]] & /@ Subsets[Range[k - 1], 4],
     -#[[1]] - #[[3]] + #[[2]] + #[[4]] & /@ Subsets[Range[k - 1], 4]}],
   Transpose[{#[[1]] + #[[3]] - #[[2]] + #[[5]] - #[[4]] & /@ Subsets[Range[k - 1], 5],
     -#[[1]] - #[[3]] + #[[2]] + #[[4]] + k - #[[5]] & /@ Subsets[Range[k - 1], 5]}],
   Transpose[{#[[1]] + #[[3]] - #[[2]] + #[[5]] - #[[4]] + k - #[[6]] & /@ Subsets[Range[k - 1], 6],
     -#[[1]] - #[[3]] + #[[2]] + #[[4]] - #[[5]] + #[[6]] & /@ Subsets[Range[k - 1], 6]}],
   Transpose[{#[[1]] + #[[3]] - #[[2]] + #[[5]] - #[[4]] + #[[7]] - #[[6]] & /@
       Subsets[Range[k - 1], 7],
     -#[[1]] - #[[3]] + #[[2]] + #[[4]] - #[[5]] + #[[6]] + k - #[[7]] & /@ Subsets[Range[k - 1], 7]}],
   Transpose[{#[[1]] + #[[3]] - #[[2]] + #[[5]] - #[[4]] + #[[7]] - #[[6]] + k - #[[8]] & /@
       Subsets[Range[k - 1], 8], -#[[1]] - #[[3]] + #[[2]] + #[[4]] -
         #[[5]] + #[[6]] + #[[8]] - #[[7]] & /@ Subsets[Range[k - 1], 8]}],
   Transpose[{#[[1]] + #[[3]] - #[[2]] + #[[5]] - #[[4]] + #[[7]] - #[[6]] + #[[9]] - #[[8]] & /@
       Subsets[Range[k - 1], 9], -#[[1]] - #[[3]] + #[[2]] + #[[4]] - #[[5]] +
         #[[6]] + #[[8]] - #[[7]] + k - #[[9]] & /@ Subsets[Range[k - 1], 9]}],
   Transpose[{#[[1]] + #[[3]] - #[[2]] + #[[5]] - #[[4]] + #[[7]] - #[[6]] + #[[9]] - #[[8]] + k - #[[10]] & /@
       Subsets[Range[k - 1], 10],
     -#[[1]] - #[[3]] + #[[2]] + #[[4]] - #[[5]] + #[[6]] + #[[8]] - #[[7]] + #[[10]] - #[[9]] & /@
       Subsets[Range[k - 1], 10]}], Transpose[
    {#[[1]] + #[[3]] - #[[2]] + #[[5]] - #[[4]] + #[[7]] - #[[6]] + #[[9]] - #[[8]] + #[[11]] - #[[10]] & /@
       Subsets[Range[k - 1], 11], -#[[1]] - #[[3]] + #[[2]] + #[[4]] - #[[5]] + #[[6]] +
         #[[8]] - #[[7]] + #[[10]] - #[[9]] + k - #[[11]] & /@ Subsets[Range[k - 1], 11]}],
   Transpose[{#[[1]] + #[[3]] - #[[2]] + #[[5]] - #[[4]] + #[[7]] - #[[6]] + #[[9]] - #[[8]] +
         #[[11]] - #[[10]] + k - #[[12]] & /@ Subsets[Range[k - 1], 12],
     -#[[1]] - #[[3]] + #[[2]] + #[[4]] - #[[5]] + #[[6]] + #[[8]] - #[[7]] + #[[10]] - #[[9]] +
         #[[12]] - #[[11]] & /@ Subsets[Range[k - 1], 12]}]}[[x]]
```

Load by branching process

Definitions: dn [i] --- the lineage size (= Z_i in the paper), L [k] --- migration load, wbar -- mean population fitness, k - number of genes in the intitial block,, W [k] -- fitness of the block of length *k.*

A single gene:

```
Clear[dn]
```

$$\text{dn}[1] = \frac{(1-\text{m})\ \text{wbar}\ (1-\text{W}[1])}{\text{wbar}-\text{W}[1]+\text{m}\,\text{W}[1]};$$

k genes:

```
dn[k_] :=
```

$$\left((-1+\text{m})\ (-1+\text{r})\left(-\text{wbar}+\text{wbar}\ \text{W}[\text{k}]-\left(\sum_{x=1}^{-1+k}\left(\sum_{i=1}^{-1+k} 2\ (1-\text{r})^{-1+k-x}\ \text{r}^{x}\ \text{Binomial}\left[-1+\text{i},\ \frac{1}{2}\ (-1+\text{x})\right]\ \text{Binomial}\left[-1-\text{i}+\text{k},\ \frac{1}{2}\ (-1+\text{x})\right]\ \text{dn}[\text{i}]\right)\right)\text{W}[\text{k}]\right)\right)\Big/$$
$$\left(-\text{wbar}+\text{r}\ \text{wbar}+(1-\text{r})^{k}\ \text{W}[\text{k}]-\text{m}\ (1-\text{r})^{k}\ \text{W}[\text{k}]\right);$$

numerical solution for the load:

```
Load[j_] := 1 - wbar /.
  FindRoot[1 - m + W[j] (1 - 1/wbar (Sum[Sum[Y[x, j, i] dn[i] r^x (1 - r)^(j-1-x), {i, 1, j - 1}],
        {x, 1, j - 1}] + dn[j] (1 - r)^(j-1))) m == wbar, {wbar, 1}]
```

Recursions for block frequencies

```
Recurs[k_] := If[k == 1, W[1]/wbar (p[1] (1 - m) + m),

  (Join[{p[1] W[1]/(wbar (1 - m)^-1) + Sum[Sum[If[j == k, p[k] (1 - m) + m, p[j] (1 - m)]
          (r^x (1 - r)^(j-1-x))/wbar (Y[x, j, 1] W[j])/1, {x, 1, j - 1}], {j, 2, k}]},
    Table[p[i] (W[i] (1 - r)^(i-1))/(wbar (1 - m)^-1) + Sum[Sum[If[j == k, p[k] (1 - m) + m, p[j] (1 - m)]
          (r^x (1 - r)^(j-1-x))/wbar (Y[x, j, i] W[j])/1, {x, 1, j - 1}], {j, i + 1, k}],
     {i, 2, k - 1}], {((p[k] (1 - m) + m) (W[k] (1 - r)^(k-1)))/wbar}])]
```

Recursions with the modifier present:

```
xRecurs[k_] :=
 Join[{(x[0] p[0] (1 - m))/wbar + p[0] Sum[x[i] (1 - m) (1 - r)^(i-1) R w[i]/wbar, {i, k}] +

    Sum[p[i] (1 - m) x[0] (1 - R) (1 - r)^(i-1) w[i]/wbar, {i, k}]},
  Table[(p[0] (1 - m) x[i] (1 - r)^(i-1) (1 - R) w[i]/wbar +
     p[i] x[0] (1 - m) R (1 - r)^(i-1) w[i]/wbar + Sum[p[0] (1 - m) x[j] w[j]/wbar
       ((1 - R) Sum[r^c (1 - r)^(j-c-1) Count[Ψ[c, j], {i, j - i}], {c, j - 1}] + R Sum[
          r^c (1 - r)^(j-c-1) Count[Ψ[c, j], {j - i, i}], {c, j - 1}]), {j, k}]), {i, k}]]
```

Invasion of modifier

Find the leading eigenvalue of the matrix of linear coefficients of block frequencies:

```
Max[Eigenvalues[Table[Coefficient[#, x[i]] & /@ xRecurs[k], {i, 0, k}]]];
```

Simulations

Simulations were peformed using Nick Barton's *Multilocus* packages: http://www.biology.ed.ac.uk/research/groups/barton/index.html Note that *Multilcous* does not seem to work in the versions of *Mathematica* above 7. In each simulation run, the population was iterated until it reached equillibrium (~1000 generations), after which the migration load and the block frequencies were estimated.

Load the required package:

```
<< NumericalModels`ExactModel`;
```

Haploid fitness:

```
Wf1[θ_, S_, n_][X_] := 1 - S + ((1 - Count[Join[X,Array[0&,Length[X]]],1]/n) S) / (1 - Count[Join[X,Array[0&,Length[X]]],1]/n θ)
```

With modifier:

```
Wf2[θ_, S_, q_, s_, n_][X_] :=
 If[X[[1]] == 1, 1 - s + ((1 - 1 / nCount[Join[Rest[X], Array[0 &, Length[X] - 1]], 1]) s) /
     (1 - 1 / nCount[Join[Rest[X], Array[0 &, Length[X] - 1]], 1] q),
   1 - S + ((1 - 1 / nCount[Join[Rest[X], Array[0 &, Length[X] - 1]], 1]) S) /
     (1 - 1 / nCount[Join[Rest[X], Array[0 &, Length[X] - 1]], 1] θ)]
```

Introgressive simulation (mean population fitness is calculated as a sum over p[i]*W[i])

Iterate a haploid population

```
Introgress[pp_, k_, m_, r_] :=
 Module[{pp1, pp0, fr, pp2},
  pp0 = MigrateExact[pp, MakePopulation[k, Array[1 &, k], 1], m];
  (*Selection*)
  fr = Flatten[MapIndexed[Wf1[θ, S, n][#1] (pp0[[1]][[#2]]) &, HaploidTypes[k]]];
  pp1 = fr / Plus @@ fr;
  pp2 =
   Drop[Plus @@ (2 pp1 (Gametes[#[[1]], #[[2]], Linkage → Array[r &, k - 1]] & /@ Table[
          {HaploidTypes[k][[1]], HaploidTypes[k][[i]]}, {i, 1, 2^k}])), 1];
  HaploidFrequencies[Join[{1 - Plus @@ pp2}, pp2], 1]]
```

To speed it up, first evaluate the following:

```
GTable =
  Table[Gametes[HaploidTypes[k][[1]], HaploidTypes[k][[i]], (k - 1) r], {i, 1, 2^k}];
```

This uses pre-defined GTable of offspring gamete distribution and so is much faster

```
IntrogressG[pp_, k_, m_, r_] :=
 Module[{pp1, pp0, fr, pp2},
  pp0 = MigrateExact[pp, MakePopulation[k, Array[1 &, k], 1], m];
  (*Selection*)
  fr = Flatten[MapIndexed[Wf1[θ, S, n][#1] (pp0[[1]][[#2]]) &, HaploidTypes[k]]];
  pp1 = fr / Plus @@ fr;
  pp2 = Drop[Plus @@ (2 pp1 GTable), 1];
  HaploidFrequencies[Join[{1 - Plus @@ pp2}, pp2], 1]]
```

IntrogressE does the same thing to Introgress, but stores wbar at the moment of selection. Uses the argument in pp = {hp, wbar} format

```
IntrogressE[pp_, k_, m_, r_] :=
 Module[{pp1, pp0, fr, pp2},
  pp0 = MigrateExact[pp[[1]], MakePopulation[k, Array[1 &, k], 1], m];
  (*Selection*)
  fr = Flatten[MapIndexed[Wf1[θ, S, n][#1] (pp0[[1]][[#2]]) &, HaploidTypes[k]]];
  pp1 = fr / (Plus @@ fr);
  pp2 =
   Drop[Plus @@ (2 pp1 (Gametes[#[[1]], #[[2]], Linkage → Array[r &, k - 1]] & /@ Table[
          {HaploidTypes[k][[1]], HaploidTypes[k][[i]]}, {i, 1, 2^k}])), 1];
  {HaploidFrequencies[Join[{1 - Plus @@ pp2}, pp2], 1], Plus @@ fr}]
```

Single Crossover Simulation. Note that recombination rate is (k-1)*r

Define the probability distribtion for gametes under single crossover and store the table for faster simulation

```
SingleGametes[X_, Y_, rho_] :=
 Plus @@ Table[Gametes[X, Y, Linkage → ReplacePart[Array[0 &, k - 1], i → rho],
     StoreResults → True], {i, k - 1}] / (k - 1)
```

```
STable = Table[
   SingleGametes[HaploidTypes[k][[1]], HaploidTypes[k][[i]], (k - 1) r], {i, 1, 2^k}];
```

The iterative function

```
IntrogressSingleR1[pp_, k_, m_] :=
 Module[{pp1, pp0, fr, pp2},
  pp0 = MigrateExact[pp, MakePopulation[k, Array[1 &, k], 1], m];
  (*Selection*)
  fr = Flatten[MapIndexed[Wf1[θ, S, n][#1] (pp0[[1]][[#2]]) &, HaploidTypes[k]]];
  pp1 = fr / (Plus @@ fr);
  pp2 = Drop[Plus @@ (2 pp1 STable), 1];
  HaploidFrequencies[Join[{1 - Plus @@ pp2}, pp2], 1]]
```

Panmictic simulation

```
hp = MakePopulation[k, Array[0 &, k]]; src = MakePopulation[k, Array[1 &, k], 1];
(* hp -- population consisting of residents only (all zeros),
 src --- the source of migrantion (all ones))
```

This is a standard way of simulating the genotype frequencies in *Multilocus.* See instructions therein. In correspondence to the numerical equations for the load and block frequencies, the order of events (migration, selection, and union of gametes) has to be chosen carefully, as well as the timepoint at which the load is measured. The following order (MigrationSelectionUnion) was used:

```
NewGametes[MakeDiploid[SelectionExact[MigrateExact[hp, src, m], Wf1[θ, S, n]]],
 Linkage → Array[r &, k - 1]]
```

The load is calculated immediately after migration:

```
1 -
 Plus @@ (MigrateExact[Nest[NewGametes[MakeDiploid[SelectionExact[MigrateExact[
            #, src, m], Wf1[θ, S, n]]], Linkage → Array[r &, k - 1]] &,
       hp, 1000], src, m][[1]] (Wf1[θ, S, n][#] & /@ HaploidTypes[k]))
```
